# The effect of different pre-treatments on sensory characteristics of unformulated pulse-based beverages

**DOI:** 10.1007/s13197-025-06303-1

**Published:** 2025-04-22

**Authors:** Neşe Yılmaz Tuncel, Havva Polat Kaya, Ali Emre Andaç, Yonca Karagül Yüceer, Necati Barış Tuncel

**Affiliations:** 1 Department of Food Technology, Faculty of Applied Sciences, Çanakkale Onsekiz Mart University, Çanakkale, 17100 Turkey; 2 Department of Food Engineering, Faculty of Engineering, Çanakkale Onsekiz Mart University, Çanakkale, 17100 Turkey

**Keywords:** Milk analogues, Plant-based milk alternatives, Legumes, Descriptive sensory analysis, Gas chromatography-olfactometry (GC-O), Off-flavour

## Abstract

The objectives of this study were to evaluate the effects of specific pre-treatments—including dry milling (control), soaking-wet milling, blanching, blanching-alkaline soaking-dehulling, vacuum treatment, and germination—on the sensory and aroma characteristics of beverages (termed as milk analogues or milk substitutes) derived from chickpea, faba bean, and cowpea as primary ingredients. In line with these objectives, solid phase micro extraction–gas chromatography–mass spectrometry (SPME–GC–MS), gas chromatography-olfactometry (GCO), and descriptive sensory evaluation were conducted, coupled with principal component analysis. Hexanal, 2-pentylfuran, 1-hexanol, and 2-heptanone were the most abundant volatiles in pulse-based milk analogues regardless of the pulse type. Among them, hexanal was found in the highest amount in all types of milk analogues. Most of the pre-treatments, including blanching, which was applied to inactivate the lipoxygenase enzymes, resulted in a significant increase in hexanal content (*p* < 0.05). However, blanching-alkaline soaking-dehulling was the most effective treatment in reducing the off-notes. Germination of 48 h or longer contributed to the development of formation of undesirable flavours.

**Supplementary Information:**

The online version contains supplementary material available at https://doi.org/10.1007/s13197-025-06303-1.

## Introduction

Given the necessity to enhance food production capacity due to population growth and the constraints of limited and polluted resources, increasing the consumption of plant-based foods emerges as one of the solutions within the framework of sustainability. Plant-based alternatives to animal products can be classified into three main groups: meat analogues, milk analogues, and egg analogues. Among these, milk analogues represent the most prevalent alternative product category, with widespread industrial production in recent years (Ignaszewski 2021). Nevertheless, plant-based milk alternatives or milk analogues are nutritionally inferior to dairy milk, especially in terms of protein content (Chalupa-Krebzdak et al. 2018). In this respect, pulses stand out as raw materials due to their high protein content and relatively low environmental impact. Yet, a notable drawback of pulse-based products is the presence of off-flavours, commonly characterized as “beany”. Any unusual taste or flavour regarded as atypical and less acceptable since consumers have a specific sensory memory of desired experiences (Chigwedere et al. 2022). Hence, there is a growing body of research on the removal of off-flavours in milk analogues, particularly those derived from pulses, and the results vary widely.

Zhang et al. (2012) investigated the effects of variety, grinding temperature, and heat processing methods on off-flavour related volatiles in soymilk. The authors found that hot grinding and UHT processes were effective methods for reducing undesirable soy odour in soymilk.

Kaczmarska et al. (2018) reported that germination and fermentation processes enhanced the consumer acceptability of lupin flour. Troszyńska et al. (2011) germinated green lentil seeds for 7 days and found that off-odour increased, while green and beany taste decreased as the germination period was prolonged, according to sensory evaluation. Xu et al. (2019) investigated the impact of germination on the flavour characteristics of chickpea, yellow pea and lentil flours, noting that the effect of germination varied depending on the pulse type. Wang et al. (2020) reported that washing with either ethanol or isopropanol was effective in removing off-flavours in pea protein enriched flour.

To our knowledge, this is the first study to investigate the effect of different pre-treatments—namely dry milling (control), soaking-wet milling, blanching, blanching-alkaline soaking-dehulling, vacuum, and germination—on the sensory characteristics of unformulated pulse-based milk analogues. To avoid any masking effects on the final products, ingredients such as sugar, gum, flavor, and stabilizers were intentionally excluded (hence, the products are referred to as unformulated). All products were produced at the same concentration, and the effects of the aforementioned pre-treatments were discussed for each product type.

## Material and methods

### Materials

Commercial chickpea (*Cicer arietinum* L.) seeds, with 9.35% crude fat, 2.99% ash, and 22.5% protein (on a dry weight basis (d.w.)), faba bean (broad bean) (*Vicia faba* L.) seeds, containing 5.10% crude fat, 3.60% ash, and 30.56% protein (d.w.), and cowpea (black-eyed pea) (*Vigna unguiculata* L.) seeds, with 3.48% crude fat, 3.71% ash, and 24.05% protein (d.w.), were sourced from supermarkets (Yılmaz Tuncel et al. 2024). To ensure better representation of the population, the same varieties of seeds were mixed prior to the pre-treatments.

### Pre-treatments

The pulses were subjected to various pre-treatments:

(a) Dry milling (control).

(b) Soaking: Dry seeds were hydrated by soaking in distilled water (1:3 w/v) at 25 °C for 24 h. Following the removal of the soaking water, the seeds underwent wet milling using fresh water.

(c) Blanching: Dry seeds were directly immersed in boiling water for 30–300 s and then immediately transferred to ice water for 2 min. The optimum blanching duration for each pulse type was established by assessing lipoxygenase (LOX) activity inhibition.

(d) Blanching-alkaline soaking-dehulling: The dry seeds were blanched in boiling water for the predetermined optimum time, then rapidly cooled in ice water. Afterward, they were soaked in alkaline water (pH 9) for 1 h to loosen the seed coat, followed by manual dehulling.

(e) Vacuum: The dry-milled seed flours were hydrated in distilled water at room temperature for 30 min on a magnetic stirrer. Afterward, the suspension underwent vacuum treatment (0.08 MPa) in a rotary evaporator at 50 °C for 30 min.

(f) Germination: The seeds underwent a 30-min steeping process in water containing 1% sodium hypochlorite (NaOCl, v/v). Afterward, they were washed under running tap water for 10 min to remove any remaining NaOCl and then soaked in distilled water at a 1:5 (w/v) ratio for 24 h to complete hydration. Germination of the hydrated seeds took place in a climate cabinet under controlled conditions of 25 °C and 95% relative humidity, with darkness maintained throughout the process. The germination process was conducted at two different levels and ended when the sprout reached a certain length. While chickpea and cowpea samples exhibited rapid germination, the faba beans required more time to germinate under the same conditions. Therefore, short term germination was designated as 24 h for chickpea and cowpea, and 48 h for faba bean. Long term germination was designated as 48 h for chickpea and cowpea, and 72 h for faba bean.

The conditions of these pre-treatments have been described in detail in the previous study (Yılmaz Tuncel et al. 2024).

### Production of the pulse-based milk substitutes

All types of milk analogues were produced at a solid to solvent ratio of 1:10 (w/v). Pre-treated and grounded pulses were extracted with water (wet-milling or dry milling followed by hydration by mixing on a magnetic stirrer at room temperature for 30 min), filtered through < 100 μm, and heated above 80 °C for starch gelatinization. After the starch hydrolysis step with a commercial α-amylase (1 μL/ g solid material, Spezyme LT-300, DuPont, Delaware, USA) according to instructions), the mixture was homogenized with Ultraturrax (IKA / T25, Staufan, Germany) at 15,000 rpm for 5 min and sterilized using an autoclave at the reference temperature of 121.1 °C for 5 min. Totally, 21 L of unformulated milk analogues (7 pre-treatment × 3 replicates (1L/batch)) were produced for each pulse type.

### Descriptive sensory analysis

The effect of the pre-treatments on the flavour profile of the pulse-based milk substitutes was investigated using descriptive sensory analysis (Meilgaard et al. 1991). First, 7 volunteer and experienced panelists (5 females, and 2 males, aged between 27 and 53 years) developed potential descriptive sensory terms on lab-scale and commercial-scale milk analogues by the moderation of the panel leader at several rounds. The developed descriptive terms, their definitions and references were given in Table 1. In the next sessions, the densities of the terms were scored using a 15-point scale (left end: no intensity, right end: strong intensity). All samples were coded with random three-digit numbers and served to panelists in plastic cups (~ 30 mL) at room temperature and under daylight. Each type of milk analogue was evaluated separately. Water and unsalted crackers were supplied to cleanse the palate in between samples.

**Table 1:** Terms with references used for descriptive sensory evaluation of the pulse-based milk analogs

Sensory terms	Description	Reference
Sweet	Taste sensation elicited by sugars	2% sucrose, solution in water = 2.0
		5% sucrose, solution in water = 5.0
Salty	Taste sensation elicited by salts	0.2% sodium chloride, solution in water = 2.5
		0,35% sodium chloride, solution in water = 5.0
Bitter	Taste sensation elicited by caffeine	0.05% caffeine, solution in water = 2.0
		0.08% caffeine, solution in water = 5.0
Sour	Taste sensation elicited by citric acid	0.05% citric acid, solution in water = 2.0
Umami	Taste sensation elicited by certain amino acids (glutamate and aspartate) and nucleotides	0.5% monosodium glutamate, solution in water = 3
		0.75% monosodium glutamate, solution in water = 4.5
Astringent	The shrinking or puckering of the tongue surface caused by substances such as tannins or alum	Brewed tea
Pulse-like (chickpea/faba bean/cowpea-like)	Aromatics associated with chickpea, faba bean and cowpea	Boiled chickpea/ faba bean/cowpea
Cooked	Aromatics associated with cooked cereals and pulses	Boiled bulgur
Sulfurous	Aromatics associated with sulfurous compounds	Boiled egg
Nutty	Aromatics associated with hazelnut/peanut	Crushed hazelnut/peanut
Earthy	Aromatic notes associated with damp soil, wet foliage or slightly undercooked potatoes	Green potato skin
Hay-like	Aromatics associated with neutral notes	Soaked oats
Boiled corn	Aromatics associated with boiled sweet corn	Canned sweet corn
Polish	Aromatics associated with polish	Oxidized flaxseed
Dirty wet towel	Aromatics associated with dirty and wet towel	Reference not used/assignment by panelist
Metallic	Aroma of minerals and metals commonly associated with metal spoon	Reference not used/assignment by panelist
Green/flower	Aromatics associated with freshly cut leaves, grass and unripe fruits	Freshly cut green grass
Fermented dough	Aromatics associated with fermented dough	Fermented dough
Medicinal	Aromatics associated with medicine	Crushed vitamin B complex

### Gas chromatography–mass spectrometry (GC–MS) analysis

Extraction of flavour compounds of the unformulated pulse-based milk substitutes (prepared at a fixed concentration of 10%) was carried out using the head space-solid phase micro extraction (HS-SPME) technique. Briefly, 5 mL of milk analogue was transferred to a 40-mL amber vial which was capped with a PTFE/silicone septa (Supelco, Belle-fonte, PA, USA). Then 1 g of NaCl and 10 µL of internal standard (IS) was added to the vial, respectively. IS was comprised of 10 µL of 2-methyl 3-heptanone in 5 mL methanol for all samples. The vial was incubated at 50 °C in water bath for 30 min. Then, SPME (2 cm to 50/30 µm DVB/Carboxen/PDMS, Supelco, Bellefonte, USA) fibre needle was inserted into the vial for 30 min to absorb volatiles at 50 °C and was injected into GC in splitless mode. The separation and identification of volatile compounds was ensured with GC–MS (GC 6890, MS 6890N, Agilent, Delaware, ABD) equipped with a HP-INNOwax column (60 m × 0.25 mm i.d. × 0.25 µm film thickness, J&W Scientific, California, USA). The mobile phase was helium gas with a flow rate of 1 mL/min. GC oven temperature was programmed from 40 °C (for 1 min) to 250 °C at 4 °C/min, with a final hold time for 10 min. The mass spectrometry was operated in the electron ionization mode at 70 eV. The interface temperature was 280 °C and mass scope was scanned from 35 to 350 m/z at a scan rate of 4.45 scan/s. The volatile compounds were identified by using National Institute of Standards and Technology (NIST) and Wiley Registry of Mass Spectral Data libraries (based on match score > 70%). Also, *n*-alkane series (C_7_–C_23_) (Sigma Aldrich, St. Louis, USA) was used as external references for the calculation of retention indices (RI) according to Kovats method (Van Den Dool & Kratz 1963).

### Gas chromatography-olfactometry (GC-O) analysis

Aroma-active compounds were extracted with the HS-SPME technique as mentioned above, without the addition of IS. Gas chromatography–olfactometry (GC-O) system was consisting of a GC-oven, flame ionization detector (FID), a non-polar column (DB-5 30 m length, 0.32 mm i.d., 0.25 μm, Agilent, Waldbronn, Germany) and a sniffing port. FID and olfactory port were maintained at temperatures 250 and 200 °C, respectively. Helium was used as the carrier gas at a flow rate of 1.7 mL/min. GC oven temperature was programmed from 40 °C (for 3 min) to 200 °C at 10 °C/min and final hold time was 10 min. GC-O procedure was run in duplicates. Aroma intensities were determined according to the post-peak intensity method using a 10-point scale (left end: 0 = no intensity, right end: 10 = strong intensity) by the sniffer who had > 300 h of experience on olfactometry technique. Odour description of the sniffer was compared with three identification methods: (1) a mixture of* n*-alkanes (C_7_–C_23_) was injected at same chromatographic conditions and the retention index (RI) (as Kovats index) of each compound was matched with reference values according to NIST database and literature, (2) the compounds were compared with those acquired by GC–MS and (3) authentic standard compounds analysed under identical chromatographic conditions. GC-O analyses were performed solely on control samples and samples which had the highest and the lowest scores in the consumer acceptability test presented in the previous study (Yılmaz Tuncel et al. 2024).

### Statistical analyses

The results of descriptive sensory analysis were evaluated with Kruskal–Wallis test and multiple comparisons were made with Dunn’s test (*p* < 0.05). The effect of the pre-treatments on the amount of the volatile components was assessed with analysis of variance (ANOVA) and significant differences within the means were compared with Tukey’s test. MINITAB (ver. 20, StatSoft, Inc., Tulsa, OK, USA) and NCSS (ver. 11, LLC, Kaysville, Utah, USA) programs were used to evaluate the data. To summarize the effect of pre-treatments, Principal Component Analysis (PCA) was performed using SPSS (version 22.0, SPSS, Chicago, IL, USA). All statistical analyses were performed on each pulse type separately. Data were presented as mean ± standard error.

## Results and discussion

### Descriptive flavour profile analysis

**Fig. 1:**
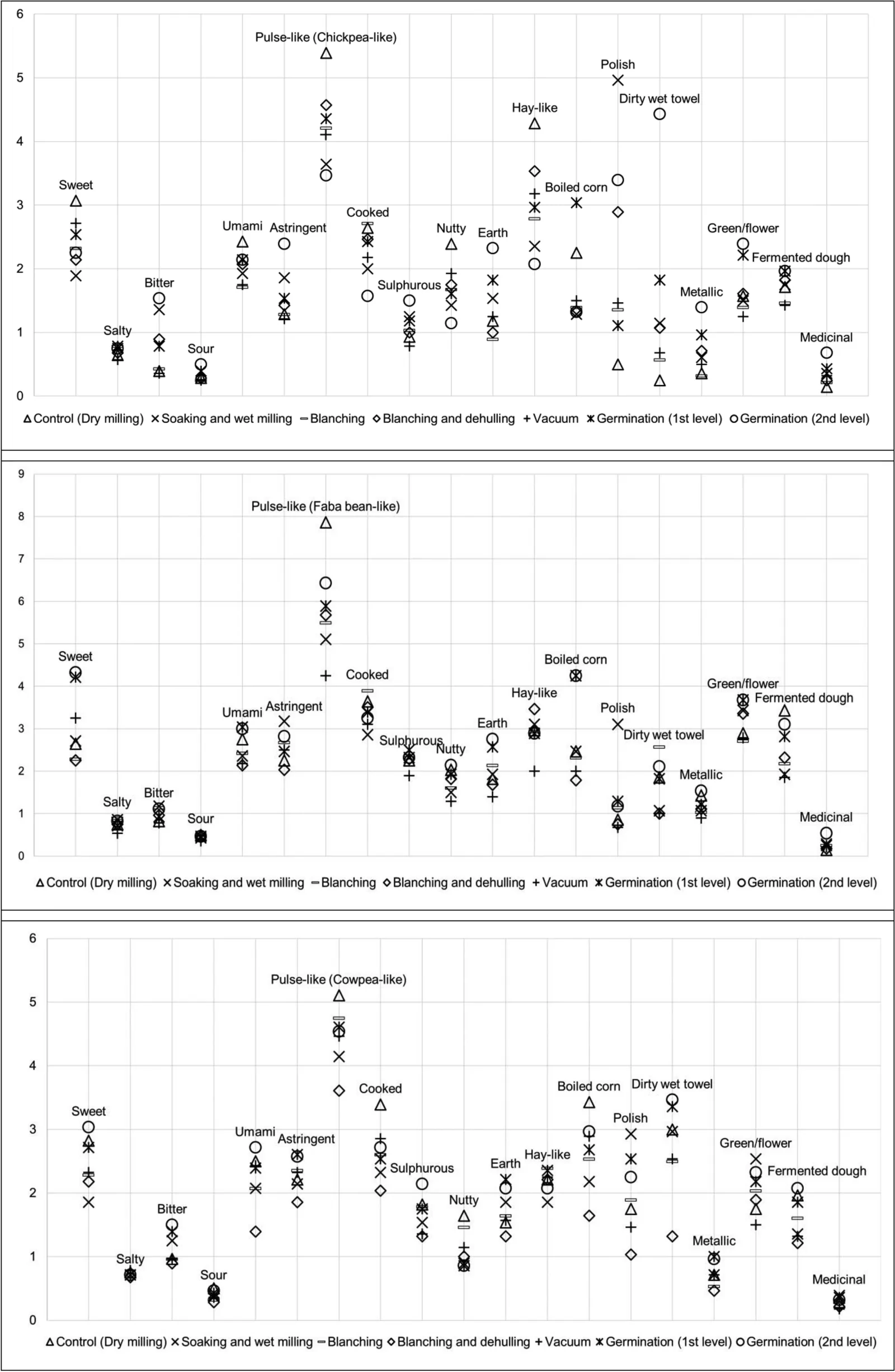
Descriptive flavor profile analysis of unformulated pulse-based milk analogs

The results of the flavour profile analysis were presented in supplementary tables (STable1, STable2, and STable3) and in Fig. 1. Five basic tastes (sweet, salty, bitter, sour, and umami) and fourteen aroma descriptors were used to characterize the flavour profile of the pulse-based milk analogues. Among them, the highest scores were given to pulse-like (chickpea-like, faba bean-like, cowpea-like) flavour for all milk analogues regardless of the pulse type (Fig. 1). This was an expected result since the products were unformulated and containing higher amounts of raw material (1:10 solid to solvent ratio) compared to their commercial counterparts. Besides, pulse-like flavour intensity was higher in control milk analogues which were not exposed to any pre-treatment. Among all, the flavours described as metallic and medicinal were found at the lowest level for any type of milk analogues. Regarding the basic tastes, sweet and umami were predominant, and the scores given for the others were below 1.5 (on 15-point scale) for any type of milk analogue. Sulfureous, earth, dirty wet towel, metallic, green, and medicinal notes were consistently found at higher levels in germinated milk analogues, particularly those subjected to long-term germination, irrespective of the pulse type. Germinated milk analogues were also the least appealed samples in consumer acceptance test (Yılmaz Tuncel et al. 2024). The panelists perceived the vacuum-treated samples as tasteless and bland. The milk analogues produced from soaked and wet-milled pulses persistently received the highest scores for polish flavour, regardless of the pulse type (Fig. 1). Although the soaking process was employed at 25 °C for 24 h, with the soaking water was discarded and hydrated seeds wet-milled using clean, fresh water, the presence of polish flavour may be attributed to microbial contamination or increased activity of degrading enzymes. The pre-treatments had an insignificant effect on the perception of cooked flavour in all samples, indirectly indicating that the blanching pre-treatment did not introduce an additional cooked flavour in the final product.

Germination process, especially long-term germination, caused a significant increase in the intensities of bitter taste and some flavours described as earth, dirty wet towel, and metallic for chickpea milk analogues (*p* < 0.05) (Supplementary Table 1). Blanching-alkaline soaking-dehulling and vacuum pre-treatments resulted in significantly lower off-flavour notes perceived as dirty wet towel, boiled popcorn, and polish in faba bean milk analogues (Supplementary Table 2). Ma et al. (2021) investigated the effect of pea milk preparation on the quality of non-dairy yoghurts and reported that dry dehulling followed by alkali water soaking markedly improved the flavour. Trindler et al. (2022) indicated that hexanal development mainly takes place in testa (seed coat) and less in cotyledons, therefore, dehulling can help to reduce potential off-flavour formation.

Another point that should be mentioned is that many of the sensory attributes such as sulfureous, green, nutty, etc. were detected by the panelists at similar intensities in all samples, whether control (untreated) or pre-treated. In other words, some flavours, desirable or undesirable, are inherent and characteristic in pulses in an agreement with Roland et al. (2017) who indicated that off-flavours inherent to the pulse can only be removed, modified, or masked, but they cannot be prevented, whereas the developed off-flavour can be limited by tuning the processing of the seeds.

### Volatiles identified by GC–MS

The amounts of the volatiles in unformulated milk analogues produced from control (untreated) and pre-treated pulses were presented in Supplementary Table 4. Aldehydes and alcohols were abundant among the volatiles. Hexanal was present in the greatest quantity in all milk analogues. The milk analogues derived from chickpeas exhibited the highest hexanal content among those produced from untreated (control) pulses. Hexanal, 1-hexanol; 1-pentanal, 2-heptanone, 2-pentylfuran, 1-pentanol, furfural, benzaldehyde, 2-furanmethanol, and benzyl alcohol were found in all milk analogues in varying amounts regardless of the pulse type. Therefore, these volatiles can be identified as the distinct flavours of the unformulated pulse-based milk analogues.

Soaking and wet milling resulted in an increase in the amount of hexanal at least twice as compared to the control (dry milled) sample, regardless of the pulse type (Supplementary Table 4). Long-term germination led to elevated levels of various volatiles associated with beany flavour, including pentanal, hexanal, and 2-heptanone, across all types of milk analogues in comparison to the control. Despite the relatively low content of lipids in pulses, beany or off-flavour is thought to be mainly derived from lipid degradation, especially oxidation of unsaturated fatty acids. LOX is the key enzyme that mainly held responsible for the enzymatic lipid oxidation (Akkad et al. 2022; Jiang et al. 2016). It was reported that the higher the activity of LOX in soybeans, the stronger the off-flavours in soymilk (Wang et al. 1997). Furthermore, it was found that soymilk with reduced off-flavour can be produced using LOX-deficient soybean varieties compared to those produced with regular soybean varieties (Yuan and Chang 2007; Kobayashi et al. 1995). Certain volatiles such as hexanal, nonanal, 2-methylfuran, 2-pentylfuran, 2-heptanone, 1-pentanol, 2-octenal were directly or indirectly associated with LOX activity in pulses (Jiang et al. 2016; Xu et al. 2019).

However, in the present study, the levels of hexanal, pentanal, 2-heptanone, 2-pentylfuran, and 1-pentanol were significantly higher in milk analogues which were produced from blanched and/or blanched-dehulled pulses when compared to their counterparts produced from untreated (control) pulses, regardless of the pulse type (*p* < 0.05). The blanching pre-treatment was intended to inactivate LOX, and a prior study has demonstrated that all pulses underwent blanching for the necessary duration to ensure LOX inactivation (Yılmaz Tuncel et al. 2024). In contrast to numerous studies in the literature, the content of the mentioned volatiles in the pulse-based milk analogues did not exhibit a correlation with the LOX activity of the pulses.

Similarly, Yuan and Chang (2007) found similar amounts of hexanal, hexanol, 1-octen-3-ol, and *trans*-2-nonenal in a regular soybean variety with a normal LOX activity (30 U/mg) and a LOX-null soybean variety with trace amounts of LOX activity (0.053 U/mg). Moreover, Jiang et al. (2016) reported that hexanal amount of microwave-treated (at 950 W) faba bean flours were higher than control flours after 1.5 min of microwave exposure, which was sufficient to totally inactivate LOX and peroxidase enzymes. The authors attributed this result to the non-enzymatic lipid autoxidation, which is a risk during heat treatments. Therefore, it can be hypothesized that non-enzymatic lipid autoxidation or other mechanisms may also be as effective as LOX-induced enzymatic oxidation in terms of promotion of beany flavour indicators and a decrease in the quantity of hexanal individually may not necessarily mean that certain amount of beany flavour or off-flavour has been removed. Furthermore, it is worth to mention that the noted volatiles do not occur individually in the products and odour characteristics of these volatiles may be different when they present in combination at various concentrations. For instance, Bott and Chambers (2006) reported that hexanal individually was not perceived as beany, however, the combination of hexanal and 1-octen-3-one resulted in the highest beany intensity among all combinations evaluated by the authors.

Among the abundant volatiles, solely 1-hexanol showed a significant decreasing trend with the pre-treatments (except for the germination) compared to control for chickpea and cowpea milk analogues. However, 1-hexanol could not be detected in control faba bean milk analogues. Nevertheless, blanching, blanching-alkaline soaking-dehulling, and vacuum pre-treatments generally resulted in significantly lower 1-hexanol levels compared to the other pre-treatments for all types of milk analogues (Supplementary Table 4). Wang et al. (1997) showed that the content of either hexanal or hexanol individually did not have a highly significant relationship with LOX activity. However, total content of hexanal and hexanol in soymilk showed a much higher correlation with the LOX activity in soybeans. The authors attributed this result to the conversion of hexanal to hexanol by catalysis of alcohol dehydrogenase (Wang et al. 1997).

Certain compounds were exclusively identified in samples subjected to long-term germination such as 1-butanol, (E)-2-hexen-1-ol, nonanal, (E)-2-octen-1-ol, and 1-hexadecanal in chickpea milk analogue; 2,3-butanedione, (E)-2-butenal, 2-ethyl-1-hexanol, and dodecanol in faba bean milk analogue; and 3-methylbutanal and (Z)-3-hexen-1-ol in cowpea milk analogue. These compounds probably formed because of the pre-treatment (germination) itself. It is widely accepted that germination lead to an increase in LOX activity and elevated levels of off-flavour in germinated pulses were attributed to the increased LOX activity (Akkad et al. 2021; Finnie et al. 2019). Yet, the results in the literature were inconclusive. For instance, Akkad et al. (2021) found that LOX activity of high tannin faba bean genotype (Fabelle) increased, while LOX activity of low tannin faba bean genotype (Snowbird) decreased as a result of germination process up to 72 h. Besides, in a previous study, it was found that LOX activities of chickpea, cowpea, and faba bean were reduced after germination (Yılmaz Tuncel et al. 2024). Conversely, the levels of volatiles linked to beany or off-flavours in milk analogues derived from germinated pulses were significantly higher than those in untreated (control) pulses. Therefore, it was concluded that the rise in off-flavour or beany notes in germinated pulses may not necessarily exhibit a positive correlation with LOX activity.

**Fig. 2:**
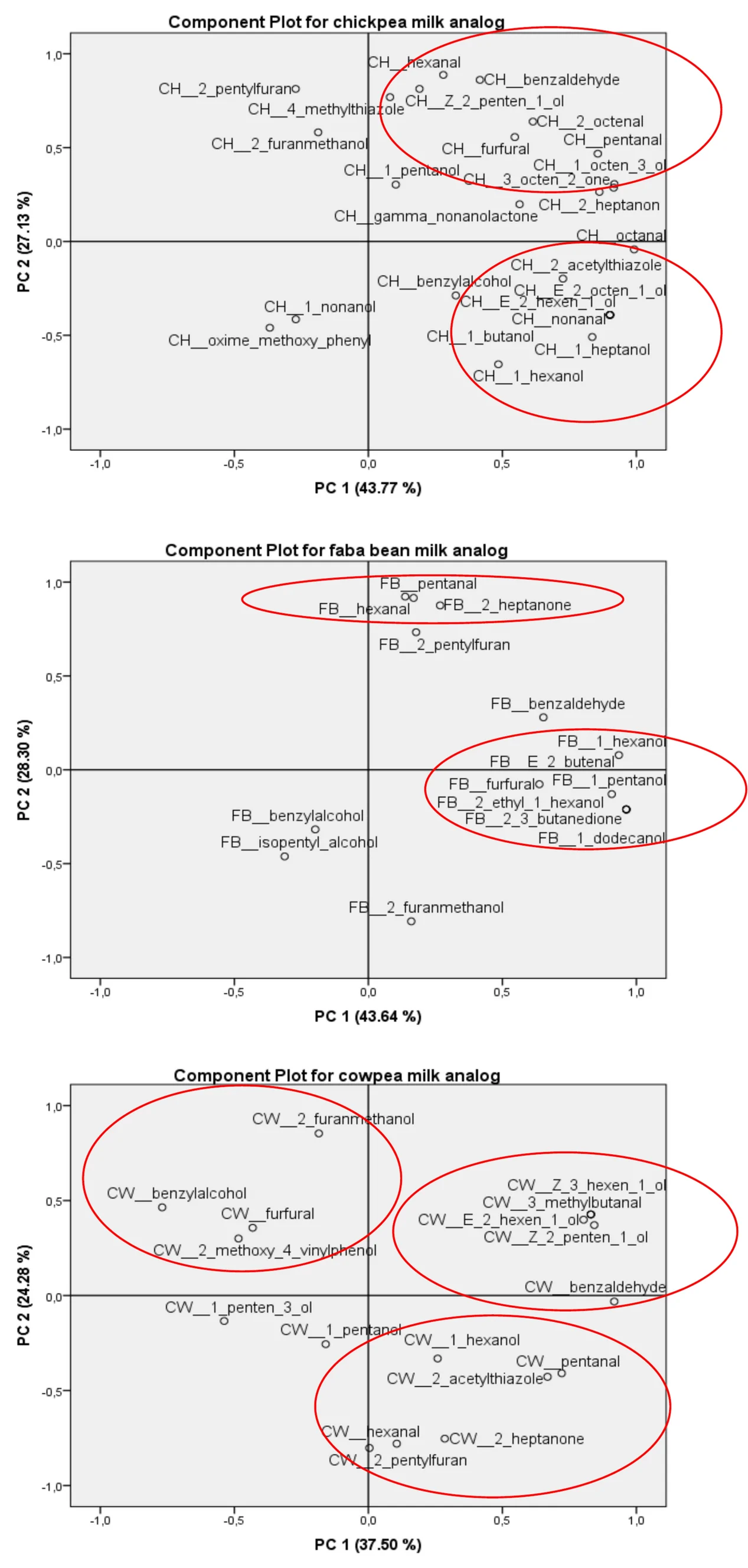
Volatiles of unformulated pulse-based milk analogs (GC–MS)

**Fig. 3:**
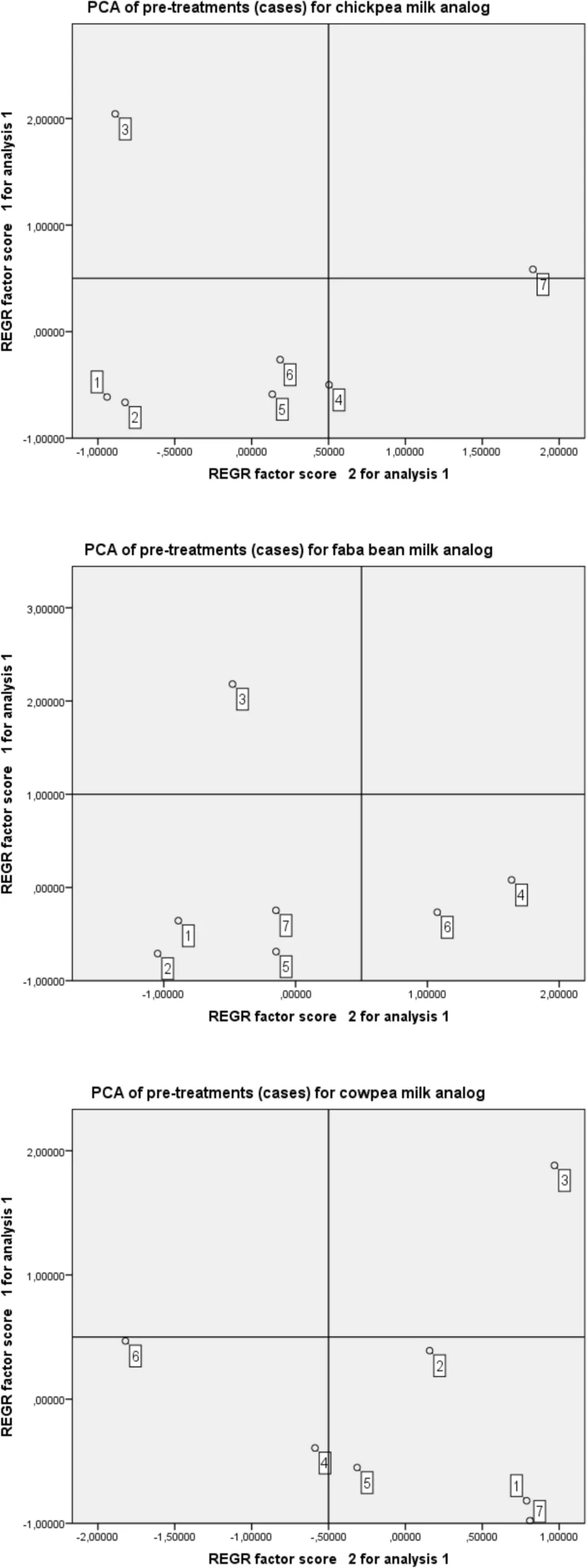
Principle component analyses (PCA) of the pre-tratments (cases) used in the production of unformulated pulse-based milk analogs (GC–MS)

Principal component analysis (PCA) was conducted to provide an overview of the clustering of volatiles (variables) (Fig. 2) and the pre-treatments (cases) (Fig. 3) based on the components’ quantities analysed by GC–MS. The most and least number of volatiles were detected in chickpea and faba bean milk analogues, respectively (Supplementary Table 4, Fig. 2). The identification of a greater number of components in chickpea milk may be associated with higher fat content in chickpeas compared to the other pulses utilized in this study.

The cluster at the first quadrant of the component plot for chickpea milk analogue represents the components which were found in vacuum-treated samples at the highest level except for 2-octenal (*p* < 0.05). The volatiles which were significantly higher in long-term germinated samples formed another cluster at the fourth quadrant (Fig. 2). Vacuum, blanching-alkaline soaking-dehulling, and long-term germination pre-treatments resulted in the highest total amount of volatile compounds, most of which were associated with off-flavour, respectively for chickpea milk analogue. It can also be seen that the noted pre-treatments were clearly separated from the others in the PCA chart built for cases (Fig. 3). Likewise, Azarnia et al. (2011) reported a significant reduction in the concentration of the volatiles in dehulled-cooked peas.

The highest total amount of volatile compounds was observed in faba bean milk analogues exposed to soaking-wet milling and blanching-alkaline soaking-dehulling pre-treatments, respectively (Supplementary Table 4). These are also the pre-treatments that resulted in the highest amount of hexanal, pentanal, and 2-heptanone which formed a cluster at the first quadrant of the component plot for faba bean milk analogue (Fig. 2). Besides, the volatiles namely 1-hexanol, (E)-2-butenal, furfural, 1-pentanol, 2-ethyl-1-hexanol, 2,3 butanedione, and 1-dodecanol, which were found significantly higher amounts in long-term germinated samples formed another cluster at the right side of PC1 (Fig. 2) (*p* < 0.05).

Cowpea milk volatiles can be categorized into 3 main clusters: volatiles found at the highest level in long-term germinated samples (quadrant 1), in vacuum-treated samples (quadrant 2), and in soaked-wet milled samples (quadrant 4) (Fig. 2). As in faba bean milk, the highest total amount of volatiles was found in soaked and wet milled cowpea milk analogues. Furthermore, the amounts of pentanal, hexanal, 2-heptanone, 2-pentylfuran, 1-hexanol, and 2-acetylthiazole, most of which are the volatiles associated with off-flavour, were significantly higher in milk analogues produced from soaked and wet milled cowpeas (p < 0.05). Ultimately, the impact of the soaking pre-treatment was more pronounced in faba bean and cowpea milk analogues compared to the chickpea milk analogue (Fig. 3).

As seen in Fig. 3, long-term germination individually stood alone as a pre-treatment for all types of milk analogues. This result shows that long-term germination caused a significant change in the volatile profile of all pulse-based milk analogues. However, descriptive sensory analysis (flavour profile analysis) indicate that this alteration is not desirable. On the other contrary, the volatile profile of milk analogues derived from pulses subjected to short-term germination and those of untreated pulses (control) were comparable and positioned in the same quadrant of the PCA chart (Fig. 3) irrespective of the pulse type, showing that the effect of the germination process on volatile profile is strongly dependent on the duration of germination. Similarly, Xu et al. (2020) found slight changes in odour characteristics of pulse protein isolates from germinated chickpea, yellow pea, and lentil after a short germination time (1 day). However, the authors reported that the overall beany-related odour sharply increased when the germination time exceeded 1 day. Besides, blanching-alkaline soaking-dehulling was distinctly separated from both the other pre-treatments and long-term germination, as depicted by the quantities of volatiles analysed by GC–MS (Fig. 3) for all types of milk analogues. Blanched and dehulled samples were also the top-rated milk analogues in the consumer acceptance test for all types of milk analogues (only second for faba bean milk analogue) (Yılmaz Tuncel et al. 2024).

### Odour active components analysed by GC-O

The combination of the data of GC–MS with GC-O, retention index (RI) value and/or the data obtained from authentic standards is highly recommended to completely profile aromatic components reliably (Xu et al. 2019). Therefore, GC-O analyses were also employed in addition to the GC–MS analyses. However, GC-O analysis was performed only in milk analogues which had the highest and the lowest scores in the consumer acceptance test (Yılmaz Tuncel et al. 2024) to simplify the interpretation. Therefore, four pre-treatments were evaluated for each pulse type: control (untreated), blanching-dehulling (high consumer acceptance), vacuum (high consumer acceptance), and long-term germination (low consumer acceptance). The average odour intensities of aroma-active compounds of pulse-based milk analogues were presented in Supplementary Table 5. Totally, 32, 25, and 24 aroma-active components were determined in chickpea, faba bean, and cowpea milk analogues, respectively in GC-O analyses. Among them, odours described as butter, green/grass, medicinal, gasoline, boiled potato, popcorn, and hay were consistently present in all types of pulse-based milk analogues and across all pre-treatments. Green/grass (hexanal) odour was found at the highest level in chickpea milk analogue in accordance with GC–MS results.

**Fig. 4:**
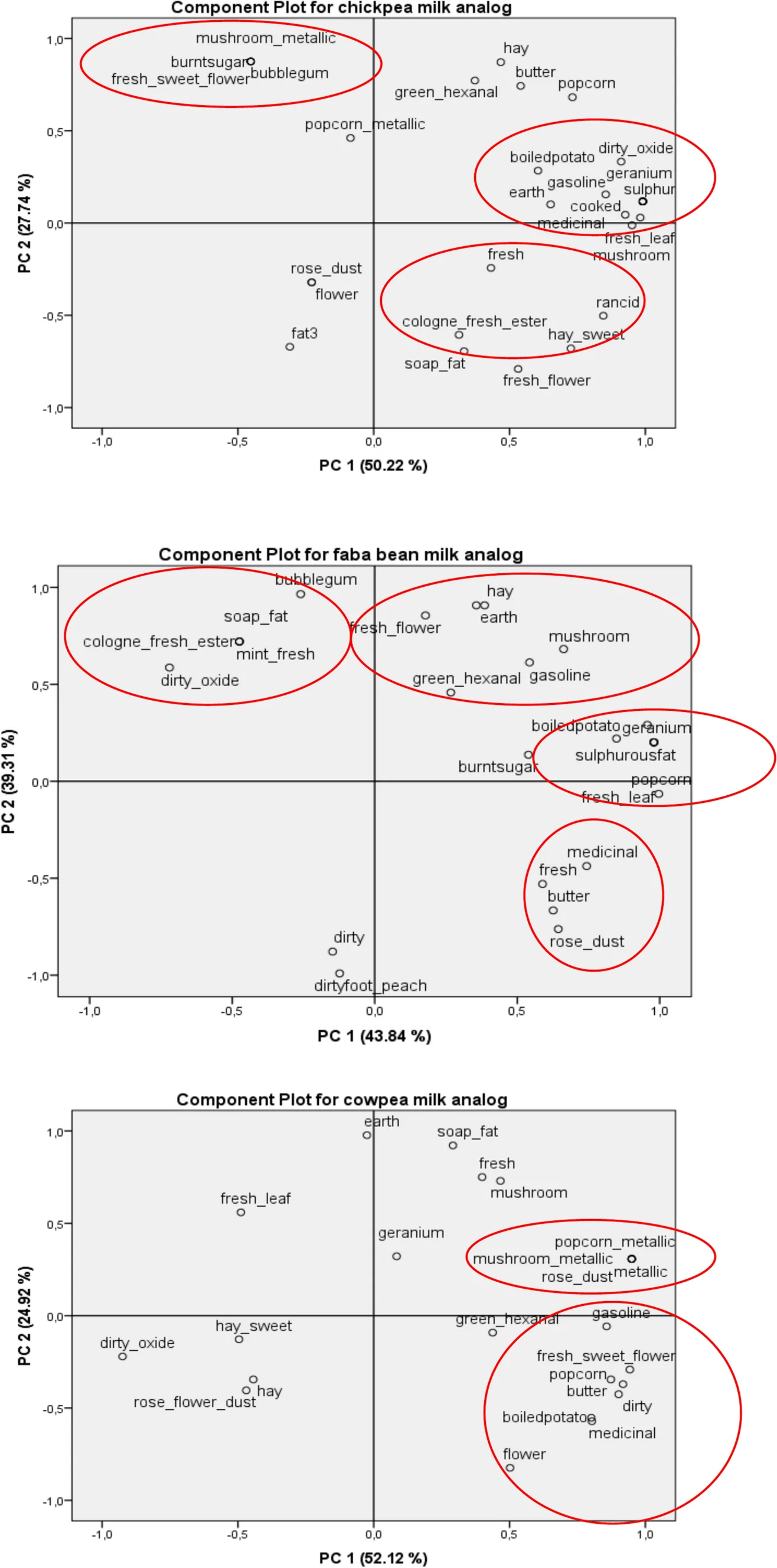
Odor active components of unformulated pulse-based milk analogs (GC-O)

**Fig. 5:**
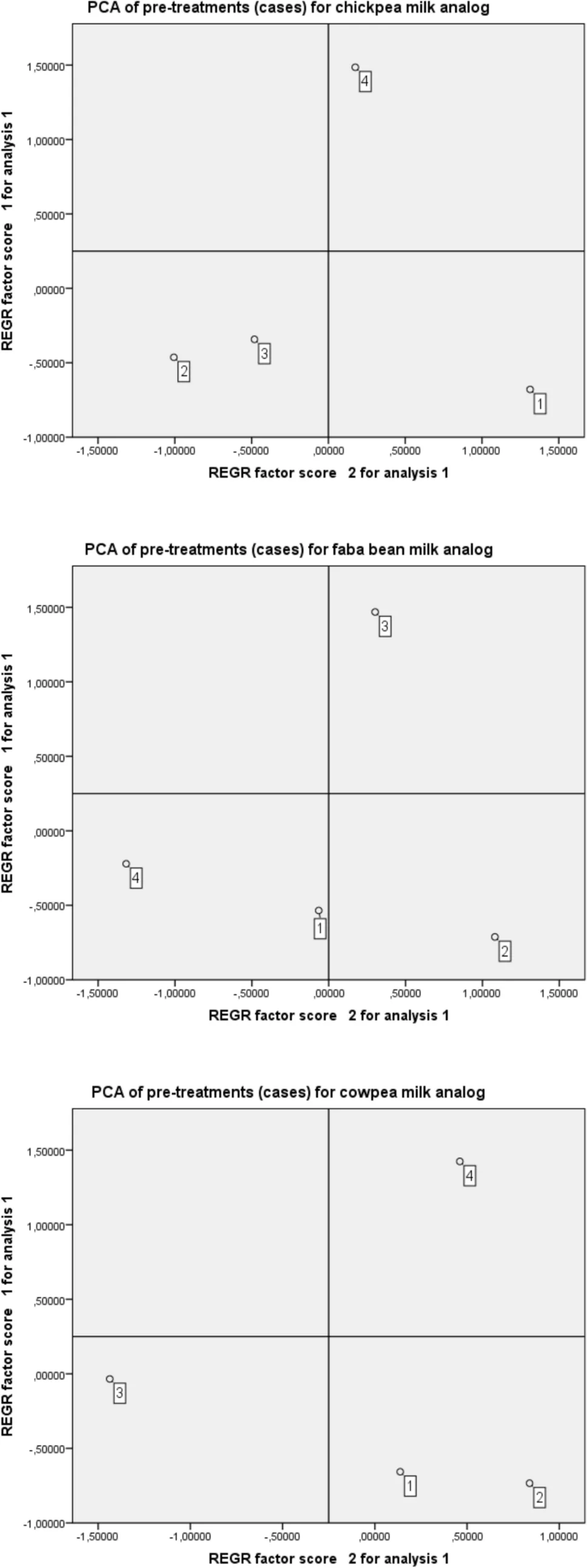
Principle component analyses (PCA) of the pre-tratments (cases) used in the production of unformulated pulse-based milk analogs (GC-O)

PCA of GC-O variables was presented in Fig. 4. It is worth to mention that gasoline, boiled potato, mushroom, geranium, dirty/oxide, cooked, medicinal and earth notes were notably higher in milk analogues produced from long-term germinated chickpeas compared to other pre-treatments (Supplementary Table 5). These odours formed a cluster at the bottom of the first quadrant on the component plot (Fig. 4.). Also, germination is clearly separated from the other pre-treatments with regard to the sensory attributes obtained from GC-O analysis for chickpea milk analogue (Fig. 5). Similarly, Xu et al. (2019) indicated increased green, beany, earthy, and grassy notes in germinated chickpea, lentil, and yellow pea flours. Moreover, Troszyńska et al. (2011) detected higher levels of off-odours in germinated lentil flours.

Odours observed in solely control (untreated) chickpea milk analogues formed another cluster at the second quadrant (Fig. 4.). Pleasant flavours such as rose/dust and flower were detected in solely vacuum-treated chickpea milk analogues. Odours which were observed in chickpea milk analogues obtained from pre-treatments other than control formed another cluster at the fourth quadrant. Hence, the flavours positioned in this cluster can be categorized as newly formed flavours due to the respective pre-treatments.

Three olfactory clusters can be formed on the right side of the component plot for faba bean milk analogues based on the pre-treatments they are exposed to: odours detected at the highest level after vacuum and blanching-dehulling (top), vacuum (middle), and vacuum and long-term germination (bottom) pre-treatments (Fig. 4). Other cluster formed at the second quadrant of the plot include the components observed at the highest level in milk analogues derived from blanched and dehulled faba beans. The odour described as dirty foot/peach was the most abundant component in faba bean milk analogues and observed at the highest level in germinated samples.

Vacuum-treated and germinated cowpea milk analogues had substantially higher levels of sensory attributes characterized as popcorn, boiled potato, and gasoline. These components were also found in vacuum-treated faba bean milk at similar levels (Supplementary Table 5). Vacuum-treatment may have eliminated some other odours and caused the aforementioned odours to be perceived more dominantly. Moreover, milk analogues produced from germinated cowpeas contained considerable levels of earth/dirty notes (Supplementary Table 5). Olfactory attributes of the milk analogues produced from germinated cowpea generally located on the right side of the PC1 on the component plot (Fig. 4). However, the cluster at the first quadrant represents the components found only in germinated cowpea milk analogues and the cluster at the fourth quadrant represents the components found in germinated and vacuum-treated cowpea milk samples at higher levels. The pre-treatments of vacuum and long-term germination yielded distinctly different sensory components compared to untreated or blanched and dehulled cowpea milk analogue with regard to GC-O analysis (Fig. 5).

## Conclusion

No pre-treatment resulted in a significant decrease in hexanal content. In fact, the hexanal amount in the milk analogues produced from pulses which were exposed to blanching to inactivate LOX, was higher than that of the milk analogues produced from untreated (control) pulses. Therefore, it was concluded that LOX activity might not necessarily be the primary factor responsible for the development of off-flavour. Moreover, upon comprehensive evaluation of all the results, it was determined that a reduction in the hexanal quantity alone may not necessarily indicate the removal of a certain amount of off-flavour. Furthermore, it was observed that each pre-treatment may cause a different effect depending on pulse type. Nonetheless, long-term germination and blanching-dehulling pre-treatments consistently differed from the other pre-treatments for all pulse types (Fig. 3). The content of the volatiles associated with beany or off- flavour such as pentanal, hexanal, and 2-heptanone was higher in milk analogues produced from germinated pulses when compared to their counterparts produced from untreated pulses, regardless of the pulse type (Supplementary Table 4). Likewise, descriptive flavour profile analysis showed that sulfureous, earth, dirty wet towel, metallic, green, and medicinal notes were detected at higher levels in long-term germinated milk analogues (Supplementary Tables 1, 2, 3). Among the pre-treatments employed in this study, long-term germination was the process that most promoted the development of undesirable off-flavours. On the other hand, blanching-dehulling emerged as a promising pre-treatment for mitigating off notes (Fig. 3, Supplementary Tables 1, 2, 3). Findings from this study can contribute to improvement of the flavour profiles of pulse-based products.

It is noteworthy that "milk analog" or "milk alternative" is commonly used to describe non-dairy products that mimic the sensory and nutritional properties of cow's milk. However, food labeling regulations in various countries prohibit the use of the term "milk" for anything other than mammal-derived products. As such, these products should more appropriately be termed pulse-based beverages.

## Supplementary Information

Below is the link to the electronic supplementary material.

Supplementary file1 (DOCX 17 KB)

Supplementary file2 (DOCX 17 KB)

Supplementary file3 (DOCX 17 KB)

Supplementary file4 (DOCX 33 KB)

Supplementary file5 (DOCX 35 KB)

## Data Availability

Data will be available upon request.
