# Supplementary material for: The effect of different pre-treatments on sensory characteristics of unformulated pulse-based beverages
Source: J Food Sci Technol. 2025 Apr 22;63(10):1927–39. doi: 10.1007/s13197-025-06303-1 (PMC13653006; doi:10.1007/s13197-025-06303-1)
Supplement: Supplementary file 1 [file 13197_2025_6303_MOESM1_ESM.docx]

| Descriptive Term | Pre-treatments | | | | | | |
| --- | --- | --- | --- | --- | --- | --- | --- |
|  | Control  (Dry milling) | Soaking-Wet milling | Blanching | Blanching-Alkaline soaking- Dehulling | Vacuum | Germination  (short-term) | Germination  (long-term) |
| Sweet | 3.07 ± 0.34 | 1.83 ± 0.34 | 2.32 ± 0.38 | 2.14 ± 0.21 | 2.71 ± 0.37 | 2.53 ± 0.47 | 2.25 ± 0.51 |
| Salty | 0.64 ± 0.14 | 0.75 ± 0.22 | 0.78 ± 0.17 | 0.67 ± 0.18 | 0.57 ± 0.10 | 0.78 ± 0.14 | 0.75 ± 0.14 |
| Bitter | 0.39 ± 0.15 ^C^ | 1.35 ± 0.15 ^AB^ | 0.42 ± 0.11 ^BC^ | 0.89 ± 0.22 ^BC^ | 0.35 ± 0.11 ^C^ | 0.78 ± 0.08 ^B^ | 1.53 ± 0.17 ^A^ |
| Sour | 0.28 ± 0.10 | 0.39 ± 0.19 | 0.25 ± 0.10 | 0.28 ± 0.10 | 0.25 ± 0.10 | 0.39 ± 0.19 | 0.50 ± 0.19 |
| Umami | 2.42 ± 0.27 | 1.92 ± 0.22 | 1.71 ± 0.22 | 2.07 ± 0.22 | 1.75 ± 0.23 | 2.14 ± 0.28 | 2.14 ± 0.26 |
| Astringent | 1.28 ± 0.29 | 1.85 ± 0.27 | 1.28 ± 0.23 | 1.42 ± 0.28 | 1.21 ± 0.30 | 1.53 ± 0.28 | 2.39 ± 0.43 |
| Pulse (Chickpea)-like | 5.39 ± 0.36 ^A^ | 3.64 ± 0.38 ^B^ | 4.21 ± 0.36 ^AB^ | 4.57 ± 0.48 ^AB^ | 4.10 ± 0.25 ^B^ | 4.35 ± 0.32 ^AB^ | 3.46 ± 0.38 ^B^ |
| Cooked | 2.64 ± 0.52 | 2.00 ± 0.34 | 2.71 ± 0.32 | 2.46 ± 0.44 | 2.17 ± 0.34 | 2.42 ± 0.32 | 1.57 ± 0.30 |
| Sulfurous | 0.92 ± 0.16 | 1.25 ± 0.23 | 1.03 ± 0.17 | 1.00 ± 0.14 | 0.78 ± 0.18 | 1.17 ± 0.21 | 1.50 ± 0.18 |
| Nutty | 2.39 ± 0.33 | 1.42 ± 0.16 | 1.67 ± 0.25 | 1.75 ± 0.30 | 1.92 ± 0.31 | 1.60 ± 0.22 | 1.14 ± 0.16 |
| Earth | 1.17 ± 0.25 ^BC^ | 1.53 ± 0.29 ^ABC^ | 0.89 ± 0.21 ^C^ | 1.00 ± 0.19 ^BC^ | 1.25 ± 0.30 ^BC^ | 1.82 ± 0.29 ^AB^ | 2.32 ± 0.39 ^A^ |
| Hay-like | 4.28 ± 0.26 ^A^ | 2.35 ± 0.35 ^CD^ | 2.78 ± 0.42 ^BCD^ | 3.53 ± 0.40 ^AB^ | 3.17 ± 0.29 ^BC^ | 2.96 ± 0.25 ^BCD^ | 2.07 ± 0.31 ^D^ |
| Boiled corn | 2.25 ± 0.33 ^AB^ | 1.28 ± 0.14 ^C^ | 1.39 ± 0.23 ^C^ | 1.32 ± 0.11 ^BC^ | 1.50 ± 0.24 ^BC^ | 3.03 ± 0.46 ^A^ | 1.32 ± 0.22 ^C^ |
| Polish | 0.50 ± 0.12 ^C^ | 4.96 ± 0.47 ^A^ | 1.35 ± 0.49 ^C^ | 2.89 ± 0.53 ^AB^ | 1.46 ± 0.49 ^C^ | 1.10 ± 0.22 ^C^ | 3.39 ± 0.60 ^AB^ |
| Dirty wet towel | 0.25 ± 0.10 ^C^ | 1.14 ± 0.26 ^B^ | 0.57 ± 0.16 ^BC^ | 1.07 ± 0.25 ^B^ | 0.67 ± 0.26 ^BC^ | 1.82 ± 0.47 ^B^ | 4.42 ± 0.43 ^A^ |
| Metallic | 0.35 ± 0.09 ^C^ | 0.60 ± 0.13 ^ABC^ | 0.32 ± 0.11 ^C^ | 0.71 ± 0.20 ^ABC^ | 0.50 ± 0.16 ^BC^ | 0.96 ± 0.23 ^AB^ | 1.39 ± 0.35 ^A^ |
| Green/flower | 1.57 ± 0.33 | 1.50 ± 0.32 | 1.39 ± 0.32 | 1.60 ± 0.36 | 1.25 ± 0.25 | 2.21 ± 0.35 | 2.39 ± 0.50 |
| Fermented dough | 1.71 ± 0.33 | 1.75 ± 0.39 | 1.46 ± 0.28 | 1.82 ± 0.35 | 1.42 ± 0.31 | 1.96 ± 0.39 | 1.96 ± 0.37 |
| Medicinal | 0.14 ± 0.09 | 0.35 ± 0.14 | 0.21 ± 0.10 | 0.25 ± 0.10 | 0.32 ± 0.14 | 0.42 ± 0.20 | 0.67 ± 0.36 |

**Supplementary Table 1.** **Flavor profile analysis of chickpea milk analog**

*Means followed by different capital letters within the same row are significantly different according to Dunn’s test (p<0.05).
