# Supplementary material for: The effect of different pre-treatments on sensory characteristics of unformulated pulse-based beverages
Source: J Food Sci Technol. 2025 Apr 22;63(10):1927–39. doi: 10.1007/s13197-025-06303-1 (PMC13653006; doi:10.1007/s13197-025-06303-1)
Supplement: Supplementary file 2 [file 13197_2025_6303_MOESM2_ESM.docx]

| Descriptive Term | Pre-treatments | | | | | | |
| --- | --- | --- | --- | --- | --- | --- | --- |
|  | Control  (Dry milling) | Soaking-Wet milling | Blanching | Blanching-Alkaline soaking- Dehulling | Vacuum | Germination  (short-term) | Germination  (long term) |
| Sweet | 2.64 ± 0.25 ^C^ | 2.71 ± 0.34 ^C^ | 2.28 ± 0.25 ^C^ | 2.25 ± 0.36 ^C^ | 3.25 ± 0.37 ^BC^ | 4.21 ± 0.35 ^AB^ | 4.32 ± 0.31 ^A^ |
| Salty | 0.75 ± 0.19 | 0.85 ± 0.19 | 0.89 ± 0.26 | 0.75 ± 0.17 | 0.53 ± 0.11 | 0.71 ± 0.14 | 0.82 ± 0.16 |
| Bitter | 0.82 ± 0.12 | 1.17 ± 0.18 | 1.14 ± 0.30 | 0.96 ± 0.17 | 0.78 ± 0.13 | 0.92 ± 0.16 | 1.10 ± 0.15 |
| Sour | 0.50 ± 0.12 | 0.42 ± 0.14 | 0.50 ± 0.18 | 0.46 ± 0.15 | 0.35 ± 0.09 | 0.46 ± 0.15 | 0.46 ± 0.15 |
| Umami | 2.75 ± 0.22 | 2.35 ± 0.34 | 2.42 ± 0.29 | 2.14 ± 0.34 | 2.17 ± 0.19 | 3.03 ± 0.31 | 3.00 ± 0.30 |
| Astringent | 2.25 ± 0.32 | 3.17 ± 0.33 | 2.67 ± 0.33 | 2.03 ± 0.30 | 2.50 ± 0.23 | 2.46 ± 0.23 | 2.82 ± 0.22 |
| Pulse (Faba bean)-like | 7.85 ± 0.20 ^A^ | 5.10 ± 0.55 ^BC^ | 5.50 ± 0.37 ^BC^ | 5.67 ± 0.37 ^BC^ | 4.25 ± 0.52 ^C^ | 5.89 ± 0.40 ^B^ | 6.42 ± 0.50 ^B^ |
| Cooked | 3.64 ± 0.16 | 2.85 ± 0.34 | 3.89 ± 0.30 | 3.50 ± 0.31 | 3.10 ± 0.22 | 3.39 ± 0.29 | 3.25 ± 0.34 |
| Sulfurous | 2.25 ± 0.24 | 2.32 ± 0.35 | 2.32 ± 0.31 | 2.28 ± 0.28 | 1.89 ± 0.31 | 2.50 ± 0.33 | 2.32 ± 0.30 |
| Nutty | 2.03 ± 0.37 | 1.50 ± 0.31 | 1.60 ± 0.29 | 1.82 ± 0.30 | 1.28 ± 0.24 | 1.92 ± 0.35 | 2.14 ± 0.47 |
| Earth | 1.82 ± 0.34 | 1.92 ± 0.37 | 2.14 ± 0.34 | 1.67 ± 0.32 | 1.39 ± 0.34 | 2.57 ± 0.41 | 2.75 ± 0.37 |
| Hay-like | 2.96 ± 0.38 | 3.10 ± 0.40 | 2.92 ± 0.37 | 3.46 ± 0.56 | 2.00 ± 0.25 | 2.89 ± 0.39 | 2.89 ± 0.37 |
| Boiled corn | 2.46 ± 0.44 ^B^ | 2.46 ± 0.76 ^B^ | 2.32 ± 0.41 ^B^ | 1.78 ± 0.28 ^B^ | 2.00 ± 0.30 ^B^ | 4.25 ± 0.73 ^A^ | 4.25 ± 0.73 ^A^ |
| Polish | 0.85 ± 0.14 ^BCD^ | 3.10 ± 0.54 ^A^ | 1.14 ± 0.23 ^BCD^ | 0.75 ± 0.10 ^CD^ | 0.67 ± 0.15 ^D^ | 1.28 ± 0.12 ^B^ | 1.17 ± 0.19 ^BC^ |
| Dirty wet towel | 1.85 ± 0.40 ^ABC^ | 1.07 ± 0.18 ^BC^ | 2.57 ± 0.39 ^A^ | 1.00 ± 0.22 ^C^ | 1.00 ± 0.21 ^C^ | 1.82 ± 0.31 ^ABC^ | 2.10 ± 0.44 ^AB^ |
| Metallic | 1.42 ± 0.27 | 1.07 ± 0.23 | 1.03 ± 0.19 | 1.10 ± 0.25 | 0.89 ± 0.16 | 1.21 ± 0.18 | 1.53 ± 0.25 |
| Green/flower | 2.89 ± 0.29 | 3.42 ± 0.54 | 2.71 ± 0.30 | 3.35 ± 0.38 | 2.78 ± 0.41 | 3.67 ± 0.40 | 3.67 ± 0.40 |
| Fermented dough | 3.42 ± 0.41 | 1.92 ± 0.30 | 2.17 ± 0.40 | 2.32 ± 0.40 | 1.85 ± 0.39 | 2.82 ± 0.53 | 3.10 ± 0.51 |
| Medicinal | 0.14 ± 0.09 | 0.28 ± 0.11 | 0.25 ± 0.11 | 0.17 ± 0.09 | 0.21 ± 1.10 | 0.25 ± 0.11 | 0.53 ± 0.35 |

**Supplementary Table 2. Flavor profile analysis of faba bean milk analog**

*Means followed by different capital letters within the same row are significantly different according to Dunn’s test (p<0.05).
