# Supplementary material for: The effect of different pre-treatments on sensory characteristics of unformulated pulse-based beverages
Source: J Food Sci Technol. 2025 Apr 22;63(10):1927–39. doi: 10.1007/s13197-025-06303-1 (PMC13653006; doi:10.1007/s13197-025-06303-1)
Supplement: Supplementary file 3 [file 13197_2025_6303_MOESM3_ESM.docx]

| Descriptive Term | Pre-treatments | | | | | | |
| --- | --- | --- | --- | --- | --- | --- | --- |
|  | Control  (Dry milling) | Soaking-Wet milling | Blanching | Blanching-Alkaline soaking- Dehulling | Vacuum | Germination  (short-term) | Germination  (long-term) |
| Sweet | 2.82 ± 0.30 | 1.85 ± 0.25 | 2.28 ± 0.18 | 2.17 ± 0.26 | 2.32 ± 0.26 | 2.71 ± 0.30 | 3.03 ± 0.38 |
| Salty | 0.75 ± 0.15 | 0.71 ± 0.17 | 0.71 ± 0.17 | 0.67 ± 0.16 | 0.78 ± 0.17 | 0.71 ± 0.15 | 0.71 ± 0.14 |
| Bitter | 0.96 ± 0.20 | 1.25 ± 0.21 | 0.96 ± 0.22 | 0.89 ± 0.19 | 0.96 ± 0.23 | 1.39 ± 0.24 | 1.50 ± 0.25 |
| Sour | 0.50 ± 0.15 | 0.39 ± 0.11 | 0.32 ± 0.11 | 0.28 ± 0.10 | 0.35 ± 0.12 | 0.42 ± 0.17 | 0.46 ± 0.17 |
| Umami | 2.50 ± 0.39 | 2.07 ± 0.17 | 2.07 ± 0.30 | 1.39 ± 0.20 | 2.42 ± 0.23 | 2.39 ± 0.38 | 2.71 ± 0.33 |
| Astringent | 2.21 ± 0.27 | 2.14 ± 0.25 | 2.35 ± 0.26 | 1.85 ± 0.29 | 2.32 ± 0.29 | 2.60 ± 0.34 | 2.57 ± 0.33 |
| Pulse (Cowpea)-like | 5.10 ± 0.54 | 4.14 ± 0.39 | 4.75 ± 0.53 | 3.60 ± 0.52 | 4.46 ± 0.56 | 4.60 ± 0.42 | 4.53 ± 0.54 |
| Cooked | 3.39 ± 0.51 | 2.32 ± 0.22 | 2.60 ± 0.29 | 2.03 ± 0.30 | 2.85 ± 0.37 | 2.53 ± 0.42 | 2.71 ± 0.29 |
| Sulfurous | 1.82 ± 0.21 | 1.53 ± 0.19 | 1.78 ± 0.30 | 1.32 ± 0.20 | 1.35 ± 0.18 | 1.75 ± 0.29 | 2.14 ± 0.32 |
| Nutty | 1.64 ± 0.40 | 0.89 ± 0.21 | 1.46 ± 0.38 | 1.00 ± 0.20 | 1.14 ± 0.28 | 0.85 ± 0.17 | 0.85 ± 0.19 |
| Earth | 1.53 ± 0.28 | 1.85 ± 0.26 | 1.64 ± 0.31 | 1.32 ± 0.32 | 1.57 ± 0.35 | 2.21 ± 0.35 | 2.07 ± 0.23 |
| Hay-like | 2.21 ± 0.24 | 1.85 ± 0.32 | 2.39 ± 0.19 | 2.21 ± 0.26 | 2.25 ± 0.29 | 2.35 ± 0.32 | 2.07 ± 0.28 |
| Boiled corn | 3.42 ± 0.38 ^A^ | 2.17 ± 0.27 ^BC^ | 2.53 ± 0.27 ^AB^ | 1.64 ± 0.28 ^C^ | 2.89 ± 0.30 ^AB^ | 2.67 ± 0.40 ^AB^ | 2.96 ± 0.31 ^AB^ |
| Polish | 1.75 ± 0.26 ^BCD^ | 2.92 ± 0.45 ^A^ | 1.89 ± 0.34 ^ABC^ | 1.03 ± 0.22 ^D^ | 1.46 ± 0.19 ^CD^ | 2.53 ± 0.21 ^AB^ | 2.25 ± 0.35 ^ABC^ |
| Dirty wet towel | 3.00 ± 0.54 ^A^ | 2.96 ± 0.48 ^A^ | 2.50 ± 0.37 ^A^ | 1.32 ± 0.32 ^B^ | 2.53 ± 0.54 ^A^ | 3.35 ± 0.64 ^A^ | 3.46 ± 0.61 ^A^ |
| Metallic | 0.71 ± 0.17 | 0.71 ± 0.16 | 0.53 ± 0.13 | 0.46 ± 0.13 | 0.71 ± 0.17 | 1.00 ± 0.22 | 0.96 ± 0.24 |
| Green/flower | 1.75 ± 0.41 | 2.53 ± 0.46 | 2.03 ± 0.24 | 1.89 ± 0.38 | 1.50 ± 0.29 | 2.17 ± 0.39 | 2.32 ± 0.44 |
| Fermented dough | 1.96 ± 0.33 | 1.35 ± 0.27 | 1.60 ± 0.28 | 1.21 ± 0.26 | 1.32 ± 0.25 | 1.85 ± 0.30 | 2.07 ± 0.31 |
| Medicinal | 0.28 ± 0.12 | 0.39 ± 0.18 | 0.28 ± 0.13 | 0.21 ± 0.10 | 0.17 ± 0.09 | 0.35 ± 0.16 | 0.32 ± 0.14 |

**Supplementary Table 3. Flavor profile analysis of cowpea milk analog**

*Means followed by different capital letters within the same row are significantly different according to Dunn’s test (p<0.05).
