# Supplementary material for: The effect of different pre-treatments on sensory characteristics of unformulated pulse-based beverages
Source: J Food Sci Technol. 2025 Apr 22;63(10):1927–39. doi: 10.1007/s13197-025-06303-1 (PMC13653006; doi:10.1007/s13197-025-06303-1)
Supplement: Supplementary file 4 [file 13197_2025_6303_MOESM4_ESM.docx]

**Supplementary Table 4. Volatile profile of unformulated pulse-based milk analogs (ng/mL) (GC-MS)**

| Unformulated chickpea milk analog | |  | Pre-treatments | | | | | | | | |
| --- | --- | --- | --- | --- | --- | --- | --- | --- | --- | --- | --- |
| Compound | Odor description | RI | Control  (Dry milling) | Soaking and  wet milling | | Blanching | Blanching –alkaline soaking-dehulling | Vacuum | | Germination  (short-term) | Germination  (long-term) |
| Pentanal | Almond, malt, pungent | 965 | 20.80 ± 4.57 ^BC^ | 25.12 ± 3.06 ^BC^ | | 28.29 ± 0.35 ^BC^ | 40.75 ± 2.14 ^B^ | 82.03 ± 0.94 ^A^ | | 13.34 ± 0.93 ^C^ | 78.79 ± 9.31 ^A^ |
| Hexanal | Green | 1058 | 349.10 ± 8.96 ^D^ | 737.24 ± 73.10 ^C^ | | 682.04 ± 36.10 ^C^ | 1146.36 ± 36.60 ^B^ | 1357.16 ± 16.30 ^A^ | | 150.96 ± 0.73 ^D^ | 680.91 ± 65.20 ^C^ |
| 1-Butanol | Medicine, fruit | 1123 | n.d. | n.d. | | n.d. | n.d. | n.d. | | n.d. | 10.87 ± 1.02 ^A^ |
| 2-Heptanone | Soap | 1168 | 30.60 ± 1.03 ^E^ | n.d. | | 44.81 ± 1.85 ^D^ | 75.18 ± 1.81 ^C^ | 127.66 ± 1.37 ^B^ | | 15.07 ± 3.00 ^F^ | 159.6 ± 6.12 ^A^ |
| 2-Pentylfuran | Green bean | 1193 | 93.18 ± 4.22 ^D^ | 219.47 ± 4.88 ^C^ | | 304.32 ± 28.00 ^B^ | 423.19 ± 1.29 ^A^ | 333.10 ± 25.00 ^B^ | | 80.78 ± 5.04 ^D^ | 65.36 ± 2.18 ^D^ |
| 1-Pentanol | Balsamic | 1222 | n.d. | 69.36 ± 8.37 ^A^ | | 38.63 ± 2.38 ^B^ | 72.92 ± 8.13 ^A^ | 24.72 ± 0.41 ^BC^ | | 14.51 ± 0.12 ^CD^ | 43.65 ± 3.84 ^B^ |
| (Z)-2-penten-1-ol | Green, plastic, rubber | 1285 | n.d. | 9.29 ± 0.94 ^B^ | | n.d. | n.d. | 18.36 ± 1.52 ^A^ | | n.d. | n.d. |
| Octanal | Fat, soap, lemon, green | 1298 | n.d. | n.d. | | n.d. | n.d. | 8.83 ± 0.28 ^B^ | | n.d. | 20.45 ± 0.33 ^A^ |
| 1-Hexanol | Resin, flower, green | 1310 | 236.56 ± 20.30 ^A^ | n.d. | | 93.72 ± 0.12 ^B^ | 89.13 ± 0.32 ^B^ | 81.68 ± 10.60 ^B^ | | 140.92 ± 7.38 ^B^ | 280.03 ± 24.50 ^A^ |
| 2-Octenal | Green | 1324 | n.d. | 33.41 ± 0.92 ^A^ | | n.d. | n.d. | n.d. | | n.d. | 13.54 ± 0.91 ^B^ |
| (E)-2-hexen-1-ol | Green, leaf, walnut | 1360 | n.d. | n.d. | | n.d. | n.d. | n.d. | | n.d. | 5.28 ± 0.31 ^A^ |
| Nonanal | Fat, citrus, green | 1383 | n.d. | n.d. | | n.d. | n.d. | n.d. | | n.d. | 14.97 ± 0.62 ^A^ |
| 3-Octen-2-one | Nut, crushed bug | 1395 | n.d. | n.d. | | n.d. | n.d. | 11.48 ± 0.33 ^A^ | | n.d. | 12.37 ± 1.02 ^A^ |
| 1-Octen-3-ol | Mushroom | 1401 | n.d. | 12.61 ± 0.63 ^B^ | | n.d. | n.d. | 30.53 ± 0.33 ^A^ | | n.d. | 34.01 ± 3.10 ^A^ |
| 1-Heptanol | Chemical, green | 1413 | 9.79 ± 0.41 ^BC^ | 14.95 ± 0.51 ^B^ | | n.d. | n.d. | n.d. | | 6.41 ± 0.47 ^BC^ | 56.85 ± 6.46 ^A^ |
| Furfural | Bread, almond, sweet | 1433 | 3.34 ± 0.43 ^BC^ | 8.76 ± 2.36 ^A^ | | 4.81 ± 0.01 ^ABC^ | 3.49 ± 0.27 ^BC^ | 8.83 ± 0.28 ^A^ | | n.d. | 7.03 ± 1.20 ^AB^ |
| Benzaldehyde | Almond, burnt sugar | 1496 | 7.31 ± 1.69 ^E^ | 14.19 ± 0.39 ^D^ | | 15.58 ± 0.23 ^D^ | 28.20 ± 1.63 ^B^ | 51.38 ± 0.32 ^A^ | | 10.43 ± 0.85 ^DE^ | 22.43 ± 1.22 ^C^ |
| (E)-2-Octen-1-ol | Soap, plastic | 1564 | n.d. | n.d. | | n.d. | n.d. | n.d. | | n.d. | 7.85 ± 0.78 ^A^ |
| 2-Furanmethanol | Burnt | 1584 | 25.35 ± 0.52 ^A^ | 11.60 ± 2.56 ^BC^ | | 16.67 ± 0.94 ^B^ | 26.60 ± 0.76 ^A^ | 27.63 ± 0.01 ^A^ | | 5.55 ± 0.96 ^C^ | 9.33 ± 1.48 ^C^ |
| 4-Methylthiazole | Roasted meat | 1600 | n.d. | n.d. | | 3.24 ± 0.13 ^B^ | n.d. | 4.97 ± 0.02 ^A^ | | n.d. | n.d. |
| 2-Acetylthiazole | Roast, nut, sulfur | 1603 | 4.59 ± 0.24 ^AB^ | n.d. | | n.d. | n.d. | 3.53 ± 0.11 ^B^ | | n.d. | 5.88 ± 0.69 ^A^ |
| 1-Nonanol | Green | 1635 | 7.04 ± 1.24 ^A^ | n.d. | | n.d. | n.d. | n.d. | | n.d. | n.d. |
| Oxime methoxy phenyl | Wet towel | 1720 | 11.04 ± 6.37 ^A^ | n.d. | | 2.19 ± 1.26 ^A^ | n.d. | n.d. | | 1.30 ± 0.75 ^A^ | n.d. |
| Benzyl alcohol | Sweet, flower | 1834 | n.d. | 6.70 ± 1.11 ^A^ | | n.d. | n.d. | n.d. | | 2.55 ± 0.32 ^B^ | 4.27 ± 0.32 ^B^ |
| 1-Hexadecanal | Cardboard | 1935 | n.d. | n.d. | | n.d. | n.d. | n.d. | | n.d. | 16.82 ± 2.07 ^A^ |
| γ- Nonalactone | Coconut, peach | 1998 | n.d. | 2.41 ± 0.08 ^A^ | | n.d. | n.d. | 1.19 ± 0.01 ^C^ | | n.d. | 1.62 ± 0.17 ^B^ |
| Unformulated faba bean milk analog | |  | Pre-treatments | | | | | | | | |
| Compound | Odor description | RI | Control  (Dry milling) | Soaking and  wet milling | | Blanching | Blanching –alkaline soaking-dehulling | Vacuum | | Germination  (short-term) | Germination  (long-term) |
| 2,3-Butanedione | Butter | 963 | n.d. | n.d. | | n.d. | n.d. | n.d. | | n.d. | 2.25±0.03 ^A^ |
| Pentanal | Almond, malt, pungent | 969 | 7.63 ± 0.51 ^C^ | 15.27 ± 3.4 ^AB^ | | 4.49 ± 0.08 ^C^ | 20.66 ± 1.62 ^A^ | 8.85 ± 1.12 ^BC^ | | 4.42 ± 1.22 ^C^ | 8.93 ± 0.05 ^BC^ |
| (E)-2-Butenal | Flower | 1055 | n.d. | n.d. | | n.d. | n.d. | n.d. | | n.d. | 2.35 ± 0.46 ^A^ |
| Hexanal | Green | 1059 | 99.8 ± 0.42 ^BC^ | 367.01 ± 1.71 ^A^ | | 71.16 ± 1.95 ^C^ | 371.47 ± 6.07 ^A^ | 122.69 ± 21.10 ^BC^ | | 74.97 ± 28.9 ^C^ | 157.56 ± 2.98 ^B^ |
| 2-Heptanone | Soap | 1167 | n.d. | 24.89 ± 0.71 ^AB^ | | 23.26 ± 9.59 ^AB^ | 50.94 ± 15.4 ^A^ | 18.68 ± 2.16 ^AB^ | | 6.33 ± 1.1 ^B^ | 24.61 ± 0.39 ^AB^ |
| Isopentyl alcohol | Malt | 1180 | n.d. | n.d. | | n.d. | n.d. | n.d. | | 8.36 ± 2.97 ^A^ | n.d. |
| 2-Pentylfuran | Green bean | 1195 | n.d. | 262.19 ± 26.4 ^A^ | | 34.53 ± 4.19 ^DE^ | 174.54 ± 37.1 ^BC^ | 223.26 ± 9.71 ^AB^ | | n.d. | 114.22 ± 5.25 ^CD^ |
| 1-Pentanol | Balsamic | 1231 | n.d. | n.d. | | n.d. | 2.70 ± 1.56 ^C^ | 8.35 ± 0.46 ^B^ | | n.d. | 17.44 ± 1.87 ^A^ |
| 1-Hexanol | Resin, flower, green | 1319 | n.d. | 98.42 ± 361 ^B^ | | 6.05 ± 62 ^C^ | 45.28 ± 1.68 ^BC^ | 30.3 ± 3.38 ^C^ | | 20.29 ± 5.15 ^C^ | 237.74 ± 0.04 ^A^ |
| Furfural | Bread, almond, sweet | 1446 | 8.22 ± 2.62 ^A^ | 2.05 ± 0.01 ^BC^ | | n.d. | 6.81 ± 1.1 ^AB^ | 6.67 ± 0.57 ^AB^ | | 2.27 ± 0.06 ^BC^ | 8.95 ± 0.34 ^A^ |
| 2-ethyl-1-hexanol | Rose, green | 1465 | n.d. | n.d. | | n.d. | n.d. | n.d. | | n.d. | 5.87 ± 0.01 ^A^ |
| Benzaldehyde | Almond, burnt sugar | 1512 | 15.13 ± 1.76 ^B^ | 5.14 ± 0.09 ^C^ | | 1.29 ± 0.37 ^C^ | 22.48 ± 0.187 ^A^ | 2.52 ± 0.46 ^C^ | | 3.43 ± 0.27 ^C^ | 20.2 ± 0.03 ^A^ |
| 2-Furanmethanol | Burnt | 1589 | 6.08 ± 0.5 ^A^ | 2.02 ± 0.12 ^BC^ | | 1.22 ± 0.23 ^BC^ | 1.08 ± 0.62 ^C^ | 3.84 ± 0.35 ^ABC^ | | 4.79 ± 1.44 ^AB^ | 4.42 ± 1.1 ^ABC^ |
| Benzyl alcohol | Sweet, flower | 1838 | 1.61 ± 0.03 ^A^ | n.d. | | n.d. | n.d. | 2.10 ± 0.41 ^A^ | | n.d. | n.d. |
| 1-Dodecanol | Wax | 1960 | n.d. | n.d. | | n.d. | n.d. | n.d. | | n.d. | 2.14 ± 0.21 ^A^ |
| Unformulated cowpea milk analog | |  | Pre-treatments | | | | | | | | |
| Compound | Odor description | RI | Control  (Dry milling) | Soaking and  wet milling | Blanching | | Blanching –alkaline soaking-dehulling | Vacuum | Germination  (short-term) | | Germination  (long-term) |
| 3-Methylbutanal | Malt | 921 | n.d. | n.d. | n.d. | | n.d. | n.d. | n.d. | | 5.81 ± 0.76 ^A^ |
| Pentanal | Almond, malt, pungent | 969 | n.d. | 15.36 ± 1.78 ^A^ | 5.96 ± 0.02 ^BC^ | | 8.05 ± 1.77 ^B^ | 2.05 ± 0.07 ^CD^ | 9.09 ± 0.41 ^B^ | | 16.52 ± 0.27 ^A^ |
| Hexanal | Green | 1061 | 60.87 ± 8.99 ^C^ | 292.01 ± 0.43 ^A^ | 64.72 ± 0.91 ^C^ | | 82.46 ± 0.34 ^BC^ | 108.56 ± 19.30 ^B^ | 62.07 ± 2.11 ^C^ | | 62.04 ± 3.21 ^C^ |
| 1-Penten-3-ol | Medicine, fruit | 1131 | n.d. | n.d. | 8.03 ± 2.15 ^A^ | | 8.20 ± 0.60 ^A^ | 5.17 ± 0.36 ^A^ | n.d. | | n.d. |
| 2-Heptanone | Soap | 1169 | n.d. | 37.42 ± 0.80 ^A^ | 26.43 ± 12.90 ^AB^ | | 37.07 ± 10.30 ^A^ | 15.23 ± 0.56 ^AB^ | 33.46 ± 2.69 ^A^ | | 19.55 ± 4.75 ^AB^ |
| 2-Pentylfuran | Green bean | 1195 | n.d. | 168.55 ± 91.2 ^A^ | 174.93 ± 88.20 ^A^ | | 93.84 ± 19.50 ^A^ | 14.79 ± 0.05 ^A^ | n.d. | | 37.52 ± 12.50 ^A^ |
| 1-Pentanol | Balsamic | 1233 | n.d. | n.d. | n.d. | | 47.52 ± 20.30 ^A^ | n.d. | 4.44 ± 2.25 ^B^ | | n.d. |
| (Z)-2-penten-1-ol | Green, plastic, rubber | 1282 | n.d. | n.d. | n.d. | | 10.94 ± 0.05 ^B^ | n.d. | 9.77 ± 0.15 ^B^ | | 33.33 ± 0.80 ^A^ |
| 1-Hexanol | Resin, flower, green | 1324 | 130.11 ± 4.06 ^B^ | 216.02 ± 1.04 ^A^ | 1.23 ± 0.18 ^D^ | | 3.71 ± 1.69 ^D^ | 17.68 ± 1.76 ^D^ | 228.35 ± 9.71 ^A^ | | 47.45 ± 3.43 ^C^ |
| (Z)-3-hexen-1-ol | Grass | 1339 | n.d. | n.d. | n.d. | | n.d. | n.d. | n.d. | | 39.57 ± 1.93 ^A^ |
| (E)-2-hexen-1-ol | Green, leaf, walnut | 1369 | n.d. | n.d. | n.d. | | n.d. | n.d. | 19.21 ± 2.16 ^A^ | | 21.48 ± 3.62 ^A^ |
| Furfural | Bread, almond, sweet | 1448 | n.d. | n.d. | n.d. | | n.d. | 2.10 ± 0.26 ^A^ | n.d. | | n.d. |
| Benzaldehyde | Almond, burnt sugar | 1526 | n.d. | 4.82 ± 0.41 ^B^ | n.d. | | n.d. | n.d. | 6.32 ± 0.26 ^A^ | | 7.38 ± 0.58 ^A^ |
| Linalool | Flower, lavender | 1566 | n.d. | n.d. | 2.44 ± 0.16 ^A^ | | n.d. | n.d. | n.d. | | 5.97 ± 0.84 ^A^ |
| 2-Furanmethanol | Burnt | 1589 | 9.50 ± 0.69 ^BC^ | n.d. | 9.18 ± 0.64 ^BC^ | | 5.05 ± 0.64 ^C^ | 14.81 ± 0.03 ^A^ | 12.92 ± 2.23 ^AB^ | | 9.98 ± 0.19 ^B^ |
| 2-Acetylthiazole | Roast, nut, sulfur | 1625 | n.d. | 5.50 ± 0.45 ^A^ | n.d. | | n.d. | 1.12 ± 0.39 ^C^ | 0.93 ± 0.28 ^C^ | | 3.58 ± 0.23 ^B^ |
| Benzyl alcohol | Sweet, flower | 1841 | 3.00 ± 0.21 ^A^ | n.d. | 0.86 ± 0.34 ^BC^ | | 0.99 ± 0.09 ^B^ | 2.25 ± 0.25 ^A^ | n.d. | | n.d. |
| 2-Methoxy-4-vinylphenol | Coconut, peach | 2136 | 4.28 ± 1.14 ^AB^ | 2.64 ± 0.00 ^ABC^ | 1.96 ± 0.33 ^BCD^ | | 1.16 ± 0.09 ^CD^ | 4.87 ± 0.61 ^A^ | n.d. | | 1.92 0.19 ^BCD^ |

* Means followed by different capital letters within each row are significantly different by Tukey (p<0.05).
