# Supplementary material for: The effect of different pre-treatments on sensory characteristics of unformulated pulse-based beverages
Source: J Food Sci Technol. 2025 Apr 22;63(10):1927–39. doi: 10.1007/s13197-025-06303-1 (PMC13653006; doi:10.1007/s13197-025-06303-1)
Supplement: Supplementary file 5 [file 13197_2025_6303_MOESM5_ESM.docx]

**Supplementary Table 5.**  **Odor active components detected in unformulated pulse-based milk analogs by GC-O**

|  | Chickpea milk analog | | | | | | | | |
| --- | --- | --- | --- | --- | --- | --- | --- | --- | --- |
| Odor description | | RI calculated | RI reference | Assigned  compound |  | Pre-treatments | | | |
|  | |  |  |  | Identification | Control (dry milling) | Blanching-alkaline soaking-dehulling | Vacuum | Germination  (long-term) |
| Butter | | 632 | 593 | 2,3-Butanedione | O, RI, MS | 1 | 0.25 | 0.8 | 1.15 |
| Sulfurous | | 705 | 711 | Methyl thiocyanate | O, RI, MS | - | - | - | 0.65 |
| Fresh/leaf | | 720 |  | Unknown | O | - | - | 0.25 | 0.9 |
| Green/grass | | 825 | 801 | Hexanal | O, RI, MS, STD | 5.5 | 4.25 | 5.25 | 5.5 |
| Mushroom/metallic | | 854 | 873 | 1-Hepten-3-one | O, RI, MS | 0.3 | - | - | - |
| Bubble gum | | 880 |  | Unknown | O | 0.4 | - | - | - |
| Medicinal | | 894 | 868 | 2-Methyl-3-furanthiol | O, RI | 1.75 | 2 | 1.5 | 3 |
| Gasoline | | 927 | 893 | Styrene | O, RI | 2 | 2.5 | - | 6 |
| Boiled potato | | 934 | 909 | Methional | O, RI, STD | 3.5 | 0.5 | 6 | 6.5 |
| Cooked | | 946 |  | Unknown | O | - | - | - | 1 |
| Popcorn | | 957 | 930 | 2-Acetyl-1-pyrroline | O, RI, STD | 5.5 | 2 | 3 | 7.5 |
| Cologne/fresh/ester | | 959 |  | Unknown | O | - | 0.4 | - | 0.25 |
| Popcorn (metallic) | | 999 | 1012 | 2-Acetylpyridine | O, RI | 4.5 | 1.5 | 5.5 | 3.5 |
| Mushroom | | 1004 | 977 | 1-octen-3-one | O, RI | 2 | 2 | 3 | 4.5 |
| Geranium | | 1010 | 983 | (Z)-1,5-octadien-3-one | O, RI | - | - | - | 2.5 |
| Fresh/Flower | | 1036 | 1039 | Benzyl alcohol | O, RI, MS | 0.6 | 1.75 | 2 | 2 |
| Rose/dust | | 1076 | 1051 | Phenylacetaldehyde | O, RI, MS | - | - | 1 | - |
| Dirty/oxide | | 1084 | 1087 | 1,2,4,5-tetramethylbenzene | O, RI | 2.25 | 0.5 | 2.5 | 6 |
| Fat (1) | | 1089 | 1100 | 3-Nonenal | O, RI | - | - | - | 3 |
| Earth | | 1126 | 1102 | 2-Ethenyl-3,5-dimethylpyrazine | O, RI | 2.5 | 3.5 | - | 5 |
| Flower | | 1176 | 1172 | (E)-Linalool oxide | O, RI | - | - | 1.5 | - |
| Fat (2) | | 1181 | 1192 | 2-Pentylpyridine | O, RI | - | - | - | 0.5 |
| Hay | | 1193 | 1162 | (E)-2-Nonenal | O, RI, MS | 4 | 1.25 | 1.5 | 4 |
| Fresh | | 1224 |  | Unknown | O | - | - | 0.75 | 0.5 |
| Rancid | | 1249 | 1243 | Phenyl cyanide | O, RI | - | 1.25 | 0.75 | 2 |
| Fat (3) | | 1255 | 1263 | 1-Decanol | O, RI, MS | - | 1.5 | - | - |
| Perfume/cologne/flower | | 1313 | 1301 | Methyl geranate | O, RI | - | - | - | 0.5 |
| Soap/fat | | 1360 | 1366 | (E)-2-undecenal | O, RI | - | 1.5 | 3 | 2 |
| Fresh/sweet/flower | | 1377 | 1393 | β-elemene | O, RI | 0.25 | - | - | - |
| Sweet/fresh/lactone | | 1394 |  | Unknown | O | - | - | - | 1.5 |
| Hay/sweet | | 1418 |  | Unknown | O | - | 0.7 | 0.5 | 0.9 |
| Burnt sugar | | 1567 |  | Unknown | O | 0.5 | - | - | - |
|  | |  |  |  |  |  |  |  |  |
|  | Faba bean milk analog | | | | | | | | |
| Odor description | | RI calculated | RI reference | Assigned  compound |  | Pre-treatments | | | |
|  | |  |  |  | Identification | Control (dry milling) | Blanching-alkaline soaking-dehulling | Vacuum | Germination  (long-term) |
| Butter | | 632 | 593 | 2,3-Butanedione | O, RI, MS | 0.5 | 0.5 | 1.75 | 2 |
| Dirty | | 691 |  | Unknown | O | - | - | - | 0.4 |
| Sulfurous | | 705 | 711 | Methyl thiocyanate | O, RI, MS | - | - | 0.25 | - |
| Fresh/leaf | | 720 |  | Unknown | O | - | - | 0.25 | - |
| Green/grass | | 825 | 701 | Hexanal | O, RI, MS, STD | 5 | 3.5 | 4.5 | 2.5 |
| Dirty foot/Peach | | 866 |  | Unknown | O | 4.5 | 2.25 | 3.25 | 6.5 |
| Bubble gum | | 880 |  | Unknown | O | 1 | 2 | 0.9 | - |
| Medicinal | | 894 | 868 | 2-Methyl-3-furanthiol | O, RI | 0.4 | 0.65 | 1.5 | 1.4 |
| Gasoline | | 927 | 893 | Styrene | O, RI | 0.8 | 4.5 | 5 | 2 |
| Boiled potato | | 934 | 909 | Methional | O, RI, STD | 3.5 | 1 | 6.5 | 0.8 |
| Popcorn | | 957 | 930 | 2-Acetyl-1-pyrroline | O, RI, STD | 3 | 2.75 | 7 | 4 |
| Cologne/fresh/ester | | 959 |  | Unknown | O | - | 0.75 | - | - |
| Mushroom | | 1004 | 977 | 1-octen-3-one | O, RI | 1 | 2 | 2.5 | 1 |
| Geranium | | 1010 | 983 | (Z)-1,5-octadien-3-one | O, RI | - | - | 2 | - |
| Fresh/Flower | | 1036 | 1039 | Benzyl alcohol | O, RI, MS | - | 1.5 | 1 | - |
| Rose/dust | | 1076 | 1051 | Phenylacetaldehyde | O, RI, MS | 0.9 | 0.25 | 1.75 | 1.9 |
| Dirty/oxide | | 1084 | 1087 | 1,2,4,5-tetramethylbenzene | O, RI | 3.5 | 3.5 | - | - |
| Burnt sugar | | 1098 | 1064 | Furaneol | O, RI | 0.4 | - | 0.4 | - |
| Earth | | 1126 | 1102 | 2-ethenyl-3,5-dimethylpyrazine | O, RI | 3 | 4 | 4.5 | - |
| Hay | | 1193 | 1162 | (E)-2-Nonenal | O, RI, MS | 1 | 1.5 | 1.5 | 0.75 |
| Fresh | | 1224 |  | Unknown | O | - | 0.5 | 1.5 | 1.75 |
| Fat | | 1255 | 1263 | 1-Decanol | O, RI, MS | - | - | 0.5 | - |
| Mint/fresh | | 1347 | 1336 | Dihydrocarvyl acetate | O, RI | - | 0.4 | - | - |
| Soap/fat | | 1360 | 1366 | (E)-2-undecenal | O, RI | - | 1 | - | - |
| Fresh/sweet/flower | | 1377 | 1393 | β-elemene | O, RI | 0.5 | 1 | 5 | 0.5 |
|  | |  |  |  |  |  |  |  |  |
|  | Cowpea milk analog | | | | | | | | |
| Odor description | | RI calculated | RI reference | Assigned compound |  | Pre-treatments | | | |
|  | |  |  |  | Identification | Control (dry milling) | Blanching-alkaline soaking-dehulling | Vacuum | Germination  (long-term) |
| Butter | | 632 | 593 | 2,3-Butanedione | O, RI, MS | 1 | 0.65 | 1.15 | 1.4 |
| Fresh/leaf | | 720 |  | Unknown | O | - | 0.4 | - | - |
| Green/grass | | 825 | 801 | Hexanal | O, RI, MS, STD | 1.25 | 3 | 3 | 3 |
| Mushroom/metallic | | 854 | 873 | 1-Hepten-3-one | O, RI, MS | - | - | - | 0.25 |
| Metallic | | 884 |  | Unknown | O | - | - | - | 0.25 |
| Medicinal | | 894 | 868 | 2-Methyl-3-furanthiol | O, RI | 0.8 | 0.75 | 2 | 2 |
| Gasoline | | 927 | 893 | Styrene | O, RI | 2.25 | 3.5 | 4 | 5 |
| Boiled potato | | 934 | 909 | Methional | O, RI, STD | 1 | 1.25 | 6 | 6 |
| Popcorn | | 957 | 930 | 2-Acetyl-1-pyrroline | O, RI, STD | 4.5 | 4.5 | 7 | 8 |
| Popcorn/metallic | | 999 | 1012 | 2-Acetylpyridine | O, RI | - | - | - | 0.5 |
| Mushroom | | 1004 | 977 | 1-octen-3-one | O, RI | 1.5 | 0.9 | - | 2 |
| Geranium | | 1010 | 983 | (Z)-1,5-octadien-3-one | O, RI | - | 2.5 | 1.25 | 1.5 |
| Rose/dust | | 1076 | 1051 | Phenylacetaldehyde | O, RI | - | - | - | 0.5 |
| Dirty/oxide | | 1084 | 1087 | 1,2,4,5-tetramethylbenzene | O, RI | 2.5 | 3.5 | 3 | - |
| Earth | | 1126 | 1102 | 2-ethenyl-3,5-dimethylpyrazine | O, RI | 5 | 5.5 | - | 5 |
| Rose/flower/dust | | 1167 |  | Unknown | O | 0.8 | - | 0.4 | - |
| Earth/dirty | | 1187 |  | Unknown | O | - | - | - | 4 |
| Hay | | 1193 | 1162 | (E)-2-Nonenal | O, RI, MS | 1.5 | 2 | 2 | 1.5 |
| Fresh | | 1224 |  | Unknown | O |  | 0.5 |  | 0.5 |
| Dirty | | 1284 |  | Unknown | O | 0.25 | - | 0.75 | 1 |
| Flower | | 1329 | 1321 | (E)-oak lactone | O, RI | - | - | 0.75 | 0.4 |
| Soap/fat | | 1360 | 1366 | (E)-2-undecenal | O, RI | 0.4 | 1 | - | 1 |
| Fresh/sweet/flower | | 1377 | 1393 | β-elemene | O, RI | 0.4 | 0.5 | 1.75 | 2.5 |
| Hay/sweet | | 1418 |  | Unknown |  | - | 1.25 | 0.9 | - |

Identification: O, odor descripted by the sniffer; RI, retention index in agreement with NIST, flavor.net; MS, Mass spectrometry data in agreement with NIST; STD, data in agreement with authentic standards.
